# General and Hygienic Treatment of Fibrinous Croup

**Published:** 1880-05

**Authors:** 


### GENERAL AND HYGIENIC TREATMENT OF FIBRINOUS CROUP.

Before the German Medical Association, held at Baden-Baden, September, 1879, Dr. C. Rauchfuss, of St. Petersburg, states that by this term he means the so-called " Laryngitis Diphtheritica," which, so far as its cause is concerned, is probably always of diphtheritic origin, but in which there is an absence of other local and constitutional signs of infection. The danger in these cases, to be sure, lies in the extension of the inflammation to the trachea, bronchi, and lungs; but this is very frequent. Were it not, we should, with tracheotomy at our command, rest comfortably assured of victory over most cases. In this discourse, however, we are not to consider the general treatment of diphtheria, but only of a fibrinous inflammation of the membrane of the larynx. Speaker gives a short sketch of the histology of the disease, and points out that it is especially the prolongations of the false membrane, the fibrinous tissue, into the orifices of the mucous follicles which form the strong attachments, and cause the adhesion between the false membrane and the mucous tissue beneath. The accumulating secretion of the mucous glands loosens and forces out these prolongations, the false membrane is undermined and cast off. We cannot but think that, if we could in practice greatly increase this secretion-pressure, and moisten and soak the membrane, we could accomplish a rapid resolution of the inflammation. This has been attempted mostly by the use of mercurials and antimonials. Speaker has used both of these for many years and with very good results. The action of the drugs in softening and loosening the membranes was so decided and prompt that, in a long list of autopsies, it was possible to pick out the cases where they had been used. Speaker now asks if we cannot attain the same results in a simpler and better way, namely: by any means which cause a great increase of mucous secretion and a soaking of the tissues. This he claims may be done by hydropathic treatment, both external and—so to speak—internal. His method is as follows: In the first place, a methodical, continuous, and large supply of water to the blood, in union with alcoholics; a mixture of water, sugar and cognac, lightly warmed, is given to the children in doses of from 100 to 200 grams every half to one hour. It is easy to give three to four litres a day, not counting other nourishment. The quantity of cognac is regulated by the age, pulse, etc., but in general is large. Besides this, the external hydropathic means are used—packings, drenchings, washings, etc., *very* frequently repeated.

R. claims that, under this treatment, all the membranes we can see externally become moist, the cardiac and respiratory action easier, and the cough looser. Who has not, after taking some warm tea or a hot punch, to "cut short a cold," experienced the benefits of the *sweating* of the membranes? Simple as it seems, R. claims that in the treatment of croup, this use of water, assisted by good alcoholics, cannot be too highly estimated.

Leaving out of view any general treatment for diphtheria or surgical interference for stenosis, his programme of treatment for a child with fibrinous croup is:

1st. Fresh air and good nourishment—few persons in the room, good ventilation, nourishment mostly with milk.

2d. The use of stimulant and hydropathic procedures to regulate the temperature, pulse, and respiration.

3d. Increasing the watery transudation upon and in the diseased membrane, and exciting increased mucous secretion by the use of the drink above described.

This use of the water is usually sufficient to keep the air of the room moist, but, if crusts or scabs form on any membranes, this moisture must be increased by steam. In connection with this, he uses disinfecting inhalations, and avoids anything mechanically or chemically irritating.

R. claims that his results by this method have been fully as good as by the mercurial treatment, though in a few severe cases he has combined the two. It, at least, has the advantage of being a carefully laid-out plan of proceeding, and guards against placing too much reliance on any one so-called "energetic" remedy.—*American Journal of Obstetrics.*

